# Proceedings of the Northern Ohio Dental Association

**Published:** 1868-05

**Authors:** Henri L. Ambler


### PROCEEDINGS OF THE NORTHERN OHIO DENTAL ASSOCIATION.

The annual meeting of the Northern Ohio Dental Association was held in the Lecture Room of Charity Hospital Medical College, May 5th, 1868, commencing at 11 o'clock, A. M. The President, Dr. B. F. Robinson, in the chair.

Minutes of the last meeting read and approved.

Members present: B. F. Robinson, W. P. Horton, C. R. Butler, Corydon Palmer, F. S. Slosson, J. F. Siddall, John Greenfield, J. E. Robinson, H. H. Newton, F. S. Whitslar, Chas. Buffett, H. L. Ambler, J. F. Galentine, C. B. Knowlton, J. G. Templeton, A. Terry, A. M. Phillips, L. Buffett, H. P. Huntington, S. B. Burnham, A. E. Lipman, B. T. Spellman.

On motion, the order of business for last year was adopted.

On motion, the examining committee were granted more time before making their report.

The election of officers for the ensuing year, resulted in the choice of the following:

*President*—W. P. Horton.

*Vice-President*—F. S. Whitslar.

*Recording Secretary*—H. L. Ambler.

*Corresponding Secretary*—Chas. Buffett.

*Treasurer*—J. E. Robinson.

*Board of Examiners*—C. R. Butler, L. Buffett, Corydon Palmer.

Dr. Robinson made a few appropriate remarks resigning his place as President, introducing his successor, Dr. Horton, who thanked the society for the honor they had conferred upon him, and trusted that he should preside in such a manner as to merit the best wishes of all. Said that since the founding of the society, it had grown rapidly, and now had as members some of the best men in the country. Hoped the bill to regulate the practice of Dentistry, would become a law, for it would do much good, helping to keep the profession and the public from being imposed upon. As regards the rubber question, we must present a united front, acting harmoniously and fight to the end. He then took the chair, and asked the pleasure of the meeting.

On motion, Drs. Taft and Kelsey were invited to sit with us, and participate in our meetings.

Dr. Buffett then read the report of the Treasurer for the past year. On motion, it was accepted and adopted.

On motion, the society adjourned until 2 P. M.

### AFTERNOON SESSION.

The society met according to adjournment. Minutes of the last meeting were read and approved.

On motion, the appointment of delegates to the American Dental Association was deferred until to-morrow.

Dr. Chas. Buffett, the committee on dentifrices reported the following formula: Prep. Chalk ℥vi.; Cuttle Bone ʒi.; Peruvian Bark ʒss.; Sugar ʒij.; Castile Soap ʒij.; Carmine, and Oil Wintergreen, *ad libitum.*

Subject for discussion, "Preservation of Exposed Dental Pulps;" remarks were made by Drs. Butler, Slosson and others. A voluntary essay was read by Dr. Spellman. Subject, "Local Hyperemia, or excess of Blood in Dental tissue."

On motion, the essay was received and placed on file.

Dr. Taft requested a copy for publication, which was granted; remarks were made upon the essay by Dr. Taft, who said it was a subject that required thought and study, in order to speak of it understandingly. He was listened to attentively, and continued some time; remarks were also made by Drs. Spellman, Butler and Buffett. The next topic for discussion was then taken under consideration, "Manipulation of Gold Foil in Filling Teeth." Dr. Butler said that Watts' crystal gold foil was first introduced in the West about ten years ago this spring, and since that time it has grown rapidly in the estimation of the profession. He said cylinder filling was a rapid method of introducing the gold; but thought that good contour fillings could not be made with them, and was not willing to be content with any one form or preparation of gold.

Dr. Palmer gave the history of the different methods of manipulating foil, and proceeded to demonstrate the same; foil should never come in contact with the hand.

Dr. Spellman thought it was not possible that the animal matter which adhered to the foil when brought in contact with the hand, could be expelled by heat; remarks were also made by Drs. Strickland, Ambler, and others.

On motion, the association adjourned until 7½ P. M.

EVENING SESSION.

The association assembled pursuant to adjournment.

Minutes of afternoon meeting read and approved.

On motion, a committee of three were appointed to investigate the professional conduct of Dr. A. E. Lyman.

The subject of manipulation of gold foil was continued; instructive remarks were made by Drs. Taft, Butler, Buffett, and others. On motion, the order of business was suspended, and Prof. Taft invited to state his views on the rubber question ; he said that our foe was not yet vanquished, that suits were being still carried on, and we must still fight them manfully; we cannot say what results may be reached in the end. On motion, it was decided that every member of this association, who has given his note for contesting with the rubber company, is legally and morally bound to pay every assessment.

On motion, the society adjourned until to-morrow at 9 P.M.

### SECOND DAY—MORNING SESSION.

The society met according to adjournment. Meeting called to order by the President, Dr. Horton, in the chair.

Minutes of last meeting read and approved.

On motion, an assessment of two dollars was levied upon each member of the society.

On motion, it was decided to hold the next annual meeting at Youngstown.

On motion, Drs. Spellman, Terry and Knowlton were appointed a committee to choose delegates to the American Dental Association, which meets at Niagara in July next.

Prof. Taft made some remarks upon the bill to regulate the practice of Dentistry in this State. He was followed by Drs. Horton, Slosson, and others.

On motion, Drs. Slosson, Horton and Butler, were appointed a committee to take the necessary measures to secure the incorporation of this society.

On motion, an order was drawn on the Treasurer to defray the expenses of the room during the sitting of the session.

On motion, the Secretary was instructed to return to

Prof. Weber, on behalf of the society, a vote of thanks for the use of his lecture room.

On motion, the Code of Dental Ethics was laid upon the table until afternoon.

The topic for discussion, Fillings of different metals approximating each other, was taken up and discussed by Drs. Butler, Slosson, Whitslar, Taft, Strickland and others.

On motion, the association adjourned until 2 P. M.

SECOND DAY—AFTERNOON SESSION.

Society met pursuant to adjournment. Minutes of morning session read and approved.

On motion, the Code of Ethics of the American Dental Association, was adopted.

On motion, the committee on incorporation were allowed further time before reporting.

The chair appointed as Executive Committee, Drs. Chas. Buffett, Strickland and Whitslar.

The committee on delegates to the American Dental Association, reported the following names: Drs. C. Buffett, B. F. Robinson, J. E. Robinson, F. S. Slosson, S. P. Huntington, H. L. Ambler and C. H. Harroun.

On motion, these delegates were appointed, and also instructed to fill any vacancy occurring in their number.

The topic, Fillings of different metals approximating each other, was then continued; remarks were made by Dr. Palmer and others.

On motion, the formula for a dentifrice presented by the committee, was adopted. Next subject for discussion, Use of the Mallet in Filling Teeth, was remarked upon by Drs. Buffett, Taft, Ambler, Whitslar and others.

Dr. Palmer then exhibited the method of placing napkins in the mouth to control the saliva, and also how an assistant could be made useful.

Dr. Butler then showed how we should use the mallet, and how to control the instrument when operating.

Improvements in Dental Mechanism was next in order for debate; remarks were made by Dr. Palmer.

Dr. Strickland cited to us something of the condition Dentistry was in thirty-three years ago, noting the valuable improvements and changes which had taken place since that time in the manufacture of Dental goods, etc.

On motion, the topic under discussion was postponed.

On motion, a vote of thanks was tendered to Prof. Taft, for his visit to the Society. Some interesting remarks [illegible] professional education were made by Prof. Taft.

The committee on Dr. Lyman's case, reported,—we have made all the investigation in said case that was in our power, considering the time at our disposal, and find, First, That he has made estimates and given his opinion as to what charges should be for Dental operations, simply by looking at the bills rendered. Second, That he has shown unfairness in carrying out and sustaining the fee bill established by this society; and further, the committee recommend that he be reprimanded.

On motion, the report was accepted and adopted. C. R. Butler, A. Terry, J. G. Templeton, committee.

On motion, Dr. Lyman was given to understand that if he still persisted in his course, that it would be sufficient cause for his expulsion at our next meeting. Dr. Lyman, standing, received the reprimand, which was administered by the President.

On motion, the association adjourned until 8 P. M.

SECOND DAY—EVENING SESSION.

Meeting called to order by the President.

On motion, reading of minutes of last meeting was postponed, and the Executive Committee instructed to appoint Essayists, and select subjects for discussion at our next meeting.

On motion, Drs. B. F. Robinson, Terry and Templeton, were appointed a committee to receive all cases of the violation of the Code of Ethics, and report the same at the next annual meeting.

Dr. Butler proceeded to remark upon the topical treatment of the Dental tissues; said the profession had great need of a Dental *materia medica*, but hoped it would not long be so. One of the most perplexing things which we have to deal with, is facial neuralgia; it has been treated locally, with aconite, chloroform, ether, and morphine, but has met with little success; local remedies only being applicable where we wish to change the tissues at a certain place. In periodontitis, the local remedies are depletion and astringents, generally making an incision, in order to derive the full benefit from our medication; then use injections of sulph. morph., aconite, belladonna, that we may produce anæsthesia of the part, and relieve the pain. In cases where there is a liability to suppurate or to formation of an abscess, we first try and reduce the inflammation by the application of cold; but not succeeding in this, use hot fomentations to hasten suppuration; hops placed in a small bag and steeped in vinegar, placed upon the face, covered with flannel, are highly useful. After opening the abscess use injections of aconite, calendula-solution carbolated iodine; also exhibited an hypodermic syringe, and explained the method of using it. In order to derive the greatest benefit it should be introduced into the cellular tissue; thought carbolic acid was better than creosote, and made it a rule to use it in all cavities before filling, never allowing it to pass the foramen at the apex of the root, without there was some disease to be reached in this way. For sensitive dentine, moisten a pledget of cotton in carbolic acid, then touch to sulph. morph., place in the cavity, letting it remain in over-night; did not think it would even destroy the pulp. Uses glycerine as a medium to convey iodine and cresote, when using for applications. In cases of exposed pulp, puts a drop of styptic colloid over it, which entirely seals it, and the pulp heals; has met with good results from teeth treated in this manner, and afterwards filled. We should be sure therapeutic agents are indicated before we prescribe or use them, and then only use those remedies that are reliable for purity, and in preparation there is generally too much dosing done, in cases of abscess and diseased antrum. Highly recommends carbolated iodine for abscess, diseased antrum, ulcers, cysts, etc., to be used as an injection. Where the gums are fungoid in growth, would dip a stick in pulverized sulph copper, and pass it around the neck of the tooth; then rinse the mouth with water to remove any excess.

The Executive Committee reported the following for our next meeting: Pathology and treatment of periodontitis, Dr. Spellman; Dental Education, Dr. Horton; Contour Fillings, Dr. Butler; Dental Hygiene, Dr. Ambler; voluntary essays by any member; report adopted and accepted.

Remarks on periodontitis were made by Drs. Butler, Templeton, and Ambler, giving local and systemic treatment, by their different methods.

Dr. Butler reported a case of a lady, æt. 60, with a fistulous opening on the outside of her face; upon examination, found the root of the inferior second bicuspid imbedded in the bone of the jaw, the alveolus being absorbed around it; the root was removed by taking a spoon excavator and cutting around it to nearly the apex, then splitting and removing it in two pieces with a hook. The fistula had been established for some months; this is a clear case of exostosis, the apex of the root being as large as a good sized bullet.

On motion, the society adjourned, closing the annual session.

HENRI L. AMBLER,
*Recording Secretary.*

